# Fault detection and classification in electrical power transmission system using artificial neural network

**DOI:** 10.1186/s40064-015-1080-x

**Published:** 2015-07-09

**Authors:** Majid Jamil, Sanjeev Kumar Sharma, Rajveer Singh

**Affiliations:** Department of Electrical Engineering, Faculty of Engineering, Jamia Millia Islamia, New Delhi, 110025 India

**Keywords:** Artificial neural networks, Feedforward networks, Back propagation algorithm, Levenberg–Marquardt algorithm

## Abstract

This paper focuses on the detection and classification of the faults on electrical power transmission line using artificial neural networks. The three phase currents and voltages of one end are taken as inputs in the proposed scheme. The feed forward neural network along with back propagation algorithm has been employed for detection and classification of the fault for analysis of each of the three phases involved in the process. A detailed analysis with varying number of hidden layers has been performed to validate the choice of the neural network. The simulation results concluded that the present method based on the neural network is efficient in detecting and classifying the faults on transmission lines with satisfactory performances. The different faults are simulated with different parameters to check the versatility of the method. The proposed method can be extended to the Distribution network of the Power System. The various simulations and analysis of signals is done in the MATLAB^®^ environment.

## Background

The electrical power system consists of so many different complex dynamic and interacting elements, which are always prone to disturbance or an electrical fault. The use of high capacity electrical generating power plants and concept of grid, i.e. synchronized electrical power plants and geographical displaced grids, required fault detection and operation of protection equipment in minimum possible time so that the power system can remain in stable condition. The faults on electrical power system transmission lines are supposed to be first detected and then be classified correctly and should be cleared in least fast as possible time. The protection system used for a transmission line can also be used to initiate the other relays to protect the power system from outages. A good fault detection system provides an effective, reliable, fast and secure way of a relaying operation.

The application of a pattern recognition technique could be useful in discriminating the faulty and healthy electrical power system. It also enables us to differentiate among three phases which phase of a three phase power system is experiencing a fault. The artificial neural networks (ANNs) are very powerful in identifying the faulty pattern and classification of fault by pattern recognition. There are lot of algorithms based upon ANN have been developed, tested and implemented practically in electrical power systems (Dalstein and Kulicke 1995; Bouthiba 2004; Venkatesan and Balamurugan 2007; Lin et al. 2001). Whei-Min Lin et al. (2001) presents the method based on pattern recognition, but the method is very complex. Angel L. Orille Fernandez et al. (2002) presented the finite impulse response (FIRANN) method to detect and classify the fault. The author uses the impulse response of voltages and currents, which limits its applications. M. S. Abdel Aziz et al. (2012), presents adaptive neuro-fuzzy inference system (ANFIS) in power distribution system. The Fourier transform is used with ANFIS, which has its inherent disadvantages. Jayabharta Reddy and Mohanta (2007) proposes and wavelet transform and fuzzy logic based algorithm for fault classification, but the fuzzy logic gives poor performance at boundary line cases. Alanzi et al. (2014) proposed the fault detection by unconventional a synchronized method, but decision making is left untouched. An efficient and reliable protection method should capable to perform more than satisfactory under various system operating conditions and different electrical network parameters. As far as ANNs are considered they exhibit excellent qualities such as normalization and generalization capability, immunity to noise, robustness and fault tolerance. Therefore, the declaration of fault made by ANN-based fault detection method should not be affected seriously by variations in various power system parameters. Therefore so many ANN-based techniques have been developed and employed in power system. The results obtained from these methods are encouraging (Kezunovic et al. 1996; Rizwan et al. 2013). Some algorithms based upon ANN for location of faults and relay architecture for protection of transmission line are also suggested by the researchers (Sanaye-Pasand and Kharashadi-Zadeh 2006; Lahiri et al. 2005). In this paper, a new algorithm based upon ANN is proposed for fast and reliable fault detection and classification. The various electrical transient system faults are modelled, simulated and an ANN based algorithm is developed for recognition of these faulty patterns. The performance of the proposed algorithm is evaluated by simulating the various types of fault and the results obtained are encouraging. It is observed that the algorithm developed is capable to perform fast and correct classification for different combinations of faulty conditions, e.g. fault type, fault resistance, fault location and short circuit MVA of the system.

The paper is divided into four categories. The section one is background, which discusses the vital points of fault detection. Second section gives over view of the artificial neural network and its training methods adopted. Third section gives the details of transmission line model and its simulation, the last fourth section is conclusion.

## Artificial neural network

Artificial neural network (ANN) can be applied to fault detection and classification effectively because it is a programming technique, capable to solve the non linear problems easily. The problems in which the information available is and in massive form can be dealt with. Also, the ANNs are able to learn with experiences, i.e. by the examples (Chaturvedi 2008). They are widely accepted and used in the problem of fault detection and fault classification because of the following features:

- Number of transmission line configuration are possible as there can be any possibility from short length, long length, single circuit transmission line to double-circuit transmission lines, etc.
- There are several methods to simulate the network with different power system conditions in a fast and reliable manner;
- The conditions of the electrical power system change after each and every disturbance. Hence a neural network is capable to incorporate the dynamic changes in the power systems.
- The ANN output is very fast, reliable and accurate depending on the training, because its working depends upon a series of very simple operations.

The algorithm which employed ANNs programming offers many advantages, but it also suffers with many disadvantages, which are very complex in nature. Some of the important factors are the selection of type of network, architecture of the network (which includes the selection of number of layers, number of neurons in each layer, selection of activation functions, learning algorithms parameters etc.), termination criteria etc. There are various parameters like values of the pre fault and post fault voltages and currents of the respective three phases in steady state required for precise fault detection and classification.

The values of the pre fault and post fault voltage and current of respective three phases are very different and are governed by the type of fault. Thus, the fault classification method required a neural network that allows it to determine the type of fault from the patterns of pre fault and post fault voltages and currents, which are generated from the values measured from a three phase transmission line of an electrical power system at one terminal. The neural network is based upon the total six number of inputs, i.e. the voltages and currents of respective three phases. The neural network is trained by using these six inputs. The total number of outputs of the neural network is four in numbers, i.e. three phases A, B, C and fourth is ground of three phase transmission line.

### Back propagation neural network (BPNN)

**Figure 1:**
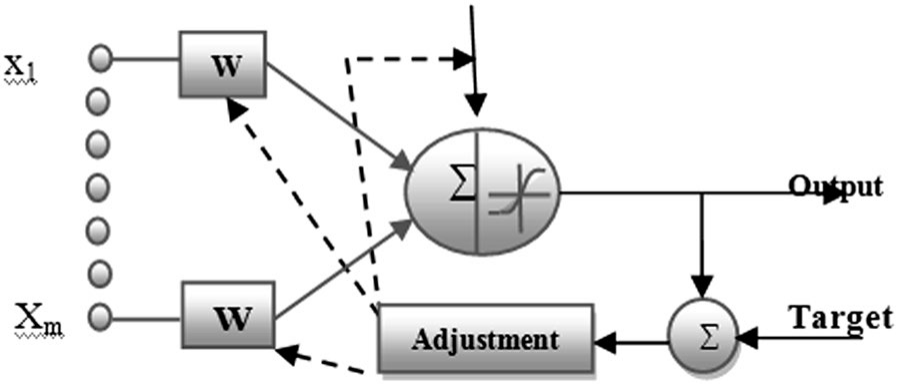
Structure of back propagation of ANN.

In the Back propagation neural network (BPNN) the output is feedback to the input to calculate the change in the values of weights. One of the major reasons for taking the back propagation algorithm is to eliminate the one of the constraints on two layers ANNs, i.e. similar inputs lead to the similar output. The error for each iteration and for each point is calculated by initiating from the last step and by sending calculated the error backwards. The weights of the back-error-propagation algorithm for the neural network are chosen randomly, feeds back in an input pair and then obtain the result. After each step, the weights are updated with the new ones and the process is repeated for entire set of inputs-outputs combinations available in the training data set provided by developer. This process is repeated until the network converges for the given values of the targets for a pre defined value of error tolerance. The entire process of back propagation can be understood by Figure 1. The back-error-propagation algorithm is effectively used for several purposes including its application to error functions (other than the sum of squared errors) and for the calculation of Jacobian and Hessian matrices. This entire process is adopted by each and every layer in the entire the network in the backward direction (Haykin 1994). The proposed algorithm uses the Mean Square Error (MSE) technique for calculating the error in each iteration.

The concept of BPNN can be understand by Figure 1.

The algorithm of BPNN is as follows:

1. Forward propagation
  $$a_{j} = \sum\limits_{i}^{m} {w_{ji}^{(1)} } x_{i}$$
  $$z_{j} = f(a_{j} )$$
  $$y_{j} = \sum\limits_{i}^{M} {w_{kj}^{(2)} } z_{j}$$
2. Output difference
  $$\delta_{k} = y_{k} - t_{k}$$
3. Back propagation for hidden layers
  $$\delta_{j} = (1 - z_{j}^{2} )\sum\limits_{k = 1}^{K} {w_{kj}^{{}} } \delta_{k}$$
4. The gradient of error with respect to first layer weights and second layer weights are calculated.
5. In this step the previous weights are updated.

where a_j_: weighted sum of inputs, w_ji_: weight associated with the connection, x_i_: inputs, z_i_: activation unit of (input) that sends a connection to unit j, δ_k_: derivative of error at kth neuron, y_i_: ith output, y_k_: activation output of unit k, t_k_: corresponding target of input and δ_j_: derivative of error wrt to a_j._

**Figure 2:**
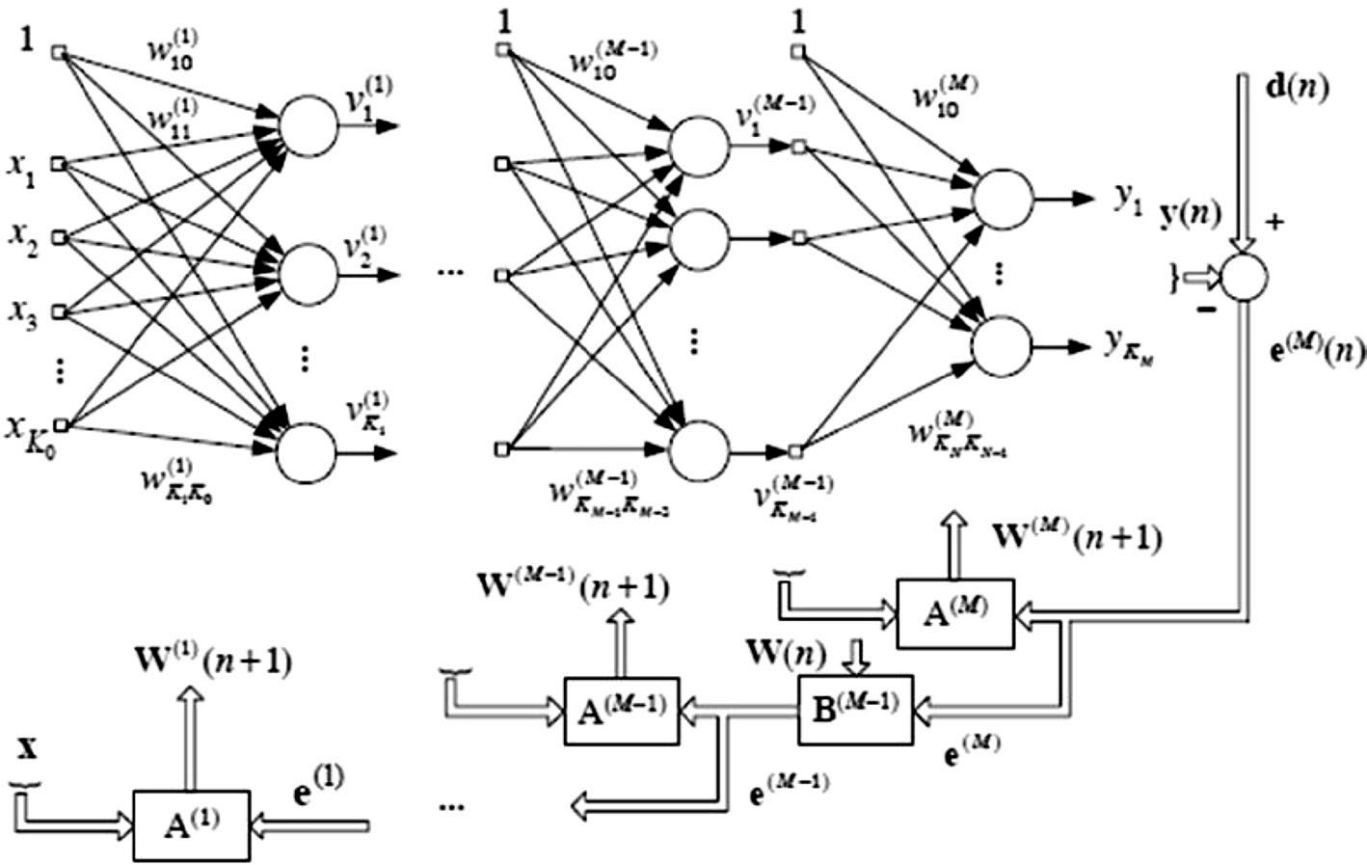
Basic structure of back-error-propagation algorithm.

The MSE for each output in each iteration is calculated by

$$MSE = \frac{1}{N}\sum\limits_{1}^{N} {\left( {E_{i} - E_{o} } \right)}^{2}$$

where *N* is number of iterations, *E*_*i*_ is actual output and *E*_*o*_ is out of the model. This entire architecture of back propagation based ANN is illustrated in Figure 2, which shows the each and every step of algorithm.

The learning rate at which the ANN is learning can be escalated by taking the optimum values of weights in very stage (Chaturvedi et al. 2004). The total numbers of iterations required for conversing the algorithm for pre define error and time taken in entire training depends upon the following factors:

- The structure of the neural network
- The size of the neural network (number of layers, etc.)
- The complexity of the problem under investigation
- The method of learning adopted (training function)
- The size of the input and output data set (training/learning patterns).

The efficiency and best performance of a developed ANN and the optimum learning method can be estimated by using the final trained network by testing with testing dataset. This testing data set is supposed to be provided by the developer and is a part of network development.

## Modelling the three phase transmission line system

A typical 400 × 10^3^ V three phase transmission line system having generators at two ends has been used for simulating, developing and implementing the developed method based upon ANNs. The system consists of two generators of 400 × 10^3^ V, each located each ends of the transmission line to simulate and study the various faults at different various locations on the transmission line.

**Figure 3:**
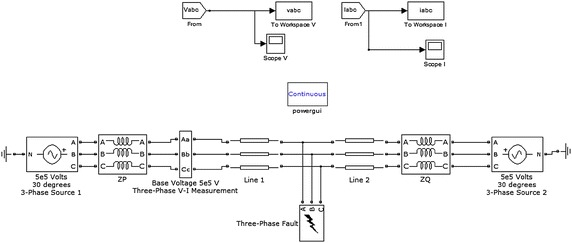
Snapshot of the studied model.

The line has been modelled using distributed type parameters, so that more accurate results can be achieved while implementing proposed scheme on very long transmission line. This power system model is being simulated by using the SimPowerSystems toolbox available in Simulink in MATLAB^®^ environment (Demuth et al. 2014). A snapshot of the model being used for studying and obtaining the training and testing 456 data sets is shown in Figure 3. Z_P_ and Z_Q_ are the source impedances of the generators on either side. The three phase V–I measurement block from the SimPowerSystem tool box is used to measure the respective three phase voltages and currents samples at the terminal A. The length of transmission line is 300 km long and the model is simulated for various types of faults at different locations along the transmission line length with different values of the fault resistances. The frequency considered for research work is 50 Hz.

### Measurement voltage and current and preprocessing of data

The three phase Voltage and current waveforms have been generated and sampled at a frequency of 1,000 Hertz. Hence, there are 20 samples per each cycle. A reduction in the overall size of the neural network improves the time performance of the neural network and this can be achieved by optimizing the feature extraction. By doing this, all of the important and relevant information present in the waveforms of the voltage and current signals can be used effectively.

**Figure 4:**
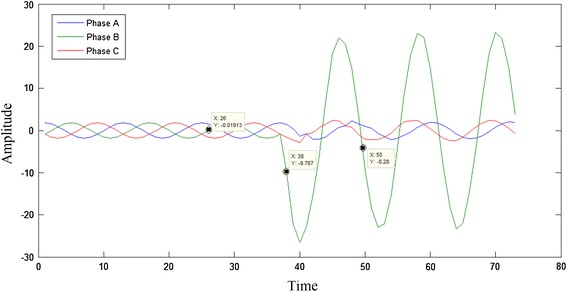
Data pre processing illustration.

Figure 4 shows the current waveform of a Phase B—ground fault at a distance of 60 km from terminal A on a 300 km transmission line. The waveform is the plot of the samples obtained at a frequency of 1,000 Hz. The inputs used to the neural network are the ratios of the voltages and currents in each of the phases before and after the occurrence of fault. The advantage of performing this scaling is to reduce the training computation time.

### Training and testing

**Figure 5:**
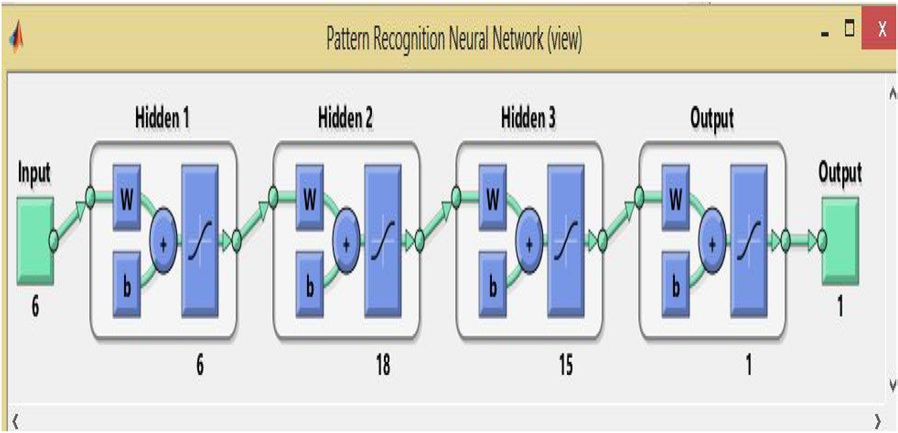
Developed BPNN model in Simulink.

Figure 5 depicts the snapshot of the developed ANN model in Simulink of MATLAB. The Neural Network toolbox in Simulink of MATLAB uses the entire data set in three parts. The first part is of date set is known as the training data set, which is used for training purpose of the neural network by computing the gradient and updating the network weights until the network converges for given value of errors. The first part is of date set is known as the validating data set and this validation dataset is used by the network during the training process (this is in the form of inputs only without assigning any outputs values) and the error in validation process for entire validating set is monitored throughout the training process. When the neural network during validation begin the over fitting the given data, the validation errors increase and when the number of validation process fails and increase beyond a particular value, the training process ends to avoid further over fitting the data and the neural network is returned to the minimum number of validation errors. The third part is testing set, the testing data set is generally not used during the training process. The third part is used to judge the overall performance of the finally developed trained neural network. If the test data set reaches up to the minimum value of mean square error at any significantly different iteration than the validation set, it means that the neural network will not be able to provide satisfactory performance and needs to be re-architecture.

For the task of training the neural networks for different stages, sequential feeding of input and output pair has been adopted. In order to obtain a large training set for efficient performance, each of the ten kinds of faults has been simulated at different locations along the considered transmission line. In view of all these issues, about 100 different fault cases for each of the 10 kinds of faults have been simulated. Apart from the type of fault, the phases that are faulted and the distance of the fault along the transmission line, the fault resistance also has been varied to include several possible real-time fault scenarios.

The fault resistance has been varied as follows: 0.25, 0.5, 0.75, 1, 5, 10, 25, 50 Ω. Also, the fault distance has been varied at an incremental factor of every 3 km on a 300 km transmission line.

### Fault detection

**Figure 6:**
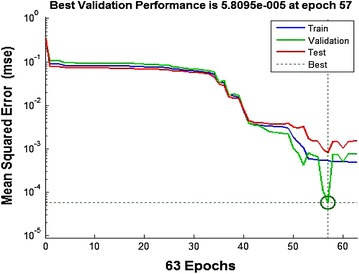
Mean-square error performance of the network.

The neural network is provided with six inputs during the fault detection process. The inputs are three voltages of respective three phases and three currents of the respective three phases. The value of input voltages and input currents are normalized with respect to the pre-fault values of the voltages and currents respectively. All ten different types of faults and no fault condition have been considered in developing the data set. The training set consist of total 8,712 input and 8,712 output samples (792 for each of the ten faults and 792 for the no fault case), which basically forms a set of six inputs and one output in each input–output pattern. The output of the neural network is in simple yes or no form, i.e. 1 or 0, which indicates whether the fault has been occurred or not. The developed architecture of artificial neural network has total five layers. Number of simulations has been carried out and a 6-10-5-3-1 neural network architecture was chosen, i.e. it has three hidden layers with 10, 5 and 3 neurons respectively (Reducindo et al. 2014; Rubio 2014; Li et al. 2014). The transfer function used for layer 1, layer 2, layer 3 and layer 4 are linear, tansig, tansig and log sig respectively, which gives best results (El-Sharkawi and Niebur 1996; Rubio et al. 2013; Ye 2014). The From the training performance plot as shown in Figure 6, it is clear that training performance shown by neural network is fine. The overall mean square error of the trained neural network is less than the pre defined value of 0.0001. The value of mean square error is 5.8095e^−005^ delivered in the end of the training of the network. Hence, this architecture is chosen as final for given input and output.

**Figure 7:**
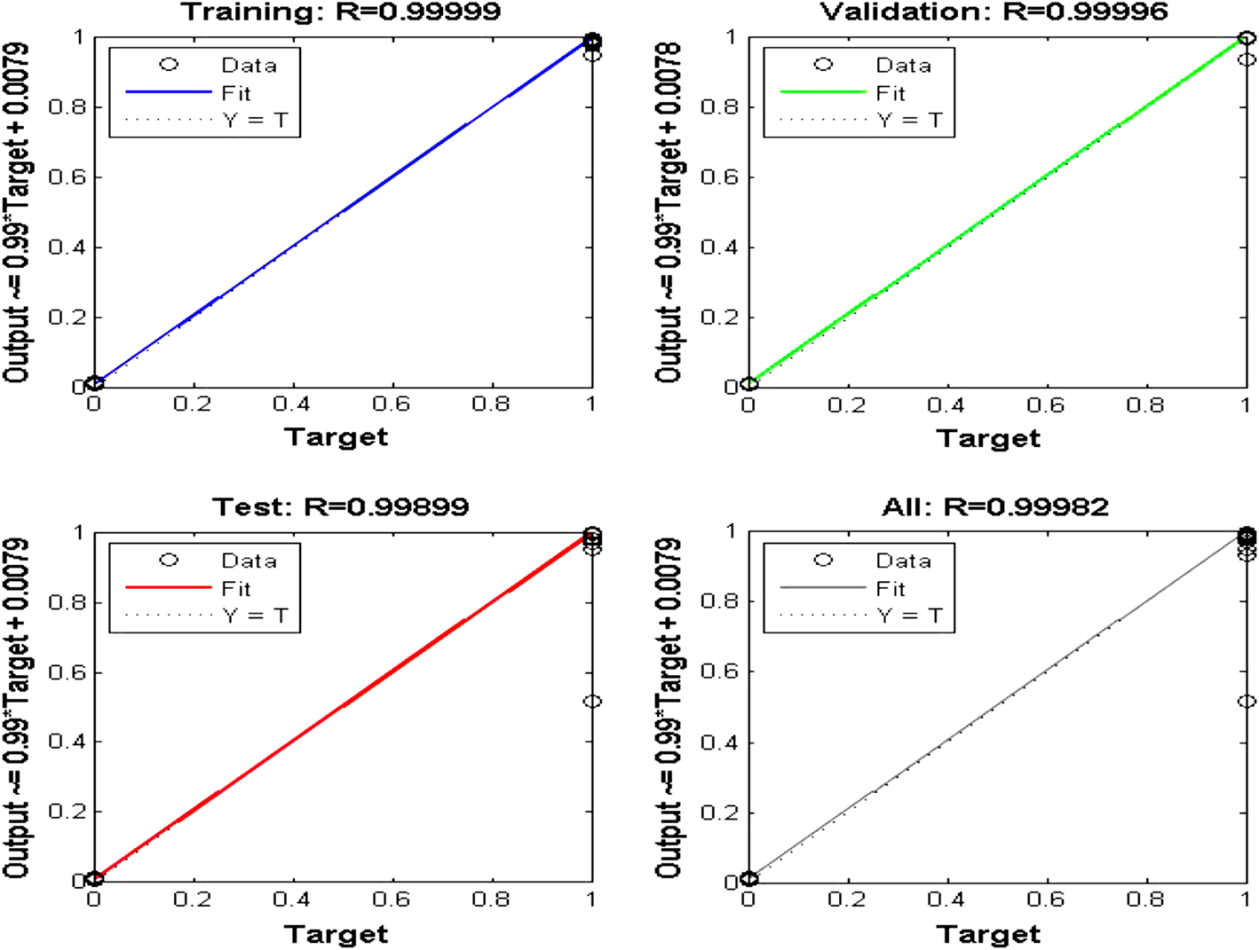
Regression FIT of the outputs vs. targets for the network.

This data set is used for training purpose the ANN. After the training of the neural network, its performance is checked by plotting the linear regression plot (available in tool box) that co-relates the targets to the outputs as shown in Figure 7.

The correlation coefficient (r) is a measure of how well the neural network’s targets can track the variations in the outputs (0 being no correlation at all and 1 being complete correlation). The correlation coefficient in this case has been found to be 0.99982 which indicates excellent correlation.

The correlation coefficient (r) is a measure of how well the neural network’s targets can track the variations in the outputs (0 being no correlation at all and 1 being complete correlation). The correlation coefficient in this case has been found to be 0.99982 which indicates excellent correlation.

**Figure 8:**
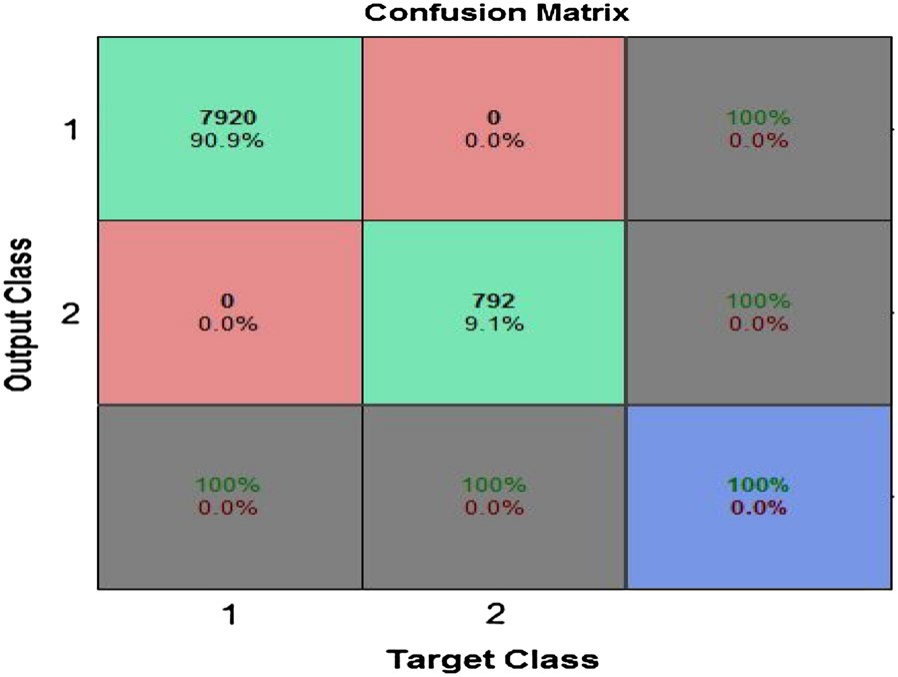
Confusion matrices for training, testing and validation phases.

Another means of testing the performance of the neural network is to plot the confusion matrices for the various types of errors that occurred for the trained neural network. Figure 8 plots the confusion matrix for the three phases of training, testing and validation. The diagonal cells in green indicate the number of cases that have been classified correctly by the neural network and the off-diagonal cells which are in red indicate the number of cases that have been wrongly classified by the ANN. The last cell in blue in each of the matrices indicates the total percentage of cases that have been classified correctly in green and the vice versa in red. It can be seen that the chosen neural network has 100 percent accuracy in fault detection.

### Fault classification

The same process that was employed in Fault Detection is also followed here in terms of the design and development of the classifier neural network. The designed network takes in the sets of six inputs as explained earlier (the three phase voltages and currents values normalized with respect to their corresponding pre-fault values). The neural network has four outputs, each of them corresponding to the fault condition of each of the three phases and one output for the ground line. Hence the outputs are either 0 or 1 denoting the absence or presence of a fault on the corresponding line (A, B, C or G where A, B and C denote the respective three phases of the transmission line system and G denotes the ground). Hence the various possible permutations can represent each of the various faults accordingly. The proposed neural network should be capable to accurately distinguish between the ten possible categories of faults. The truth table representing the faults and the ideal output for each of the faults is illustrated in Table 1.

**Table 1:** Fault classifier ANN outputs for various faults

Type of fault	Phase A	Phase B	Phase C	Ground
AG	1	0	0	1
BG	0	1	0	1
CG	0	0	1	1
AB	1	1	0	0
BC	0	1	1	0
CA	1	0	1	0
ABG	1	1	0	1
BCG	0	1	1	1
CAG	1	0	1	1
ABC	1	1	1	0

The training set contains total 7,920 inputs and output pattern (792 for each type of fault out of ten faults) with six inputs and one output in each input–output combination. Back-propagation networks with a variety of combinations of hidden layers and the different number of neurons in each hidden layer were analyzed. Of those, the one that achieved satisfactory performance was the neural network 6-38-4, i.e. 6 neurons in the input layer, 1 hidden layer with 38 neurons in it and four neurons in the output layer.

**Figure 9:**
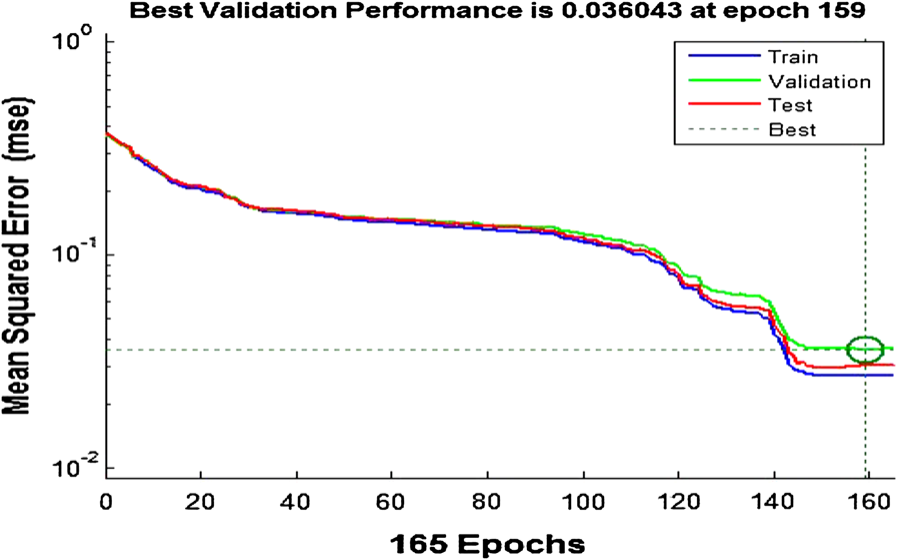
Mean-square error performance of the network.

The overall mean square error of the trained neural network is 0.036043 and it can be seen from Figure 9 that the testing and the validation curves have similar characteristics which is an indication of efficient training.

**Figure 10:**
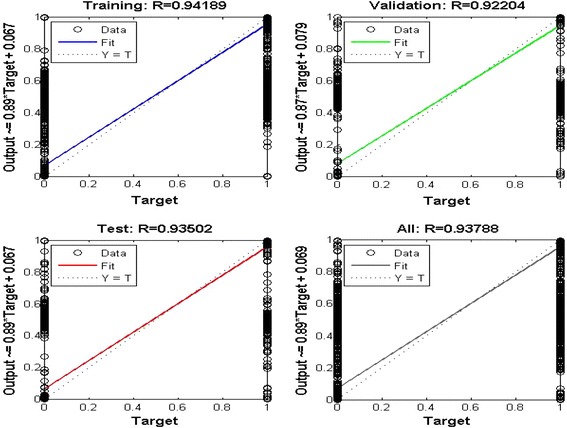
Curve of regression Fit for the outputs vs. targets of the proposed ANN.

The performance of the trained neural network is tested in two ways, i.e. first by plotting the linear regression that relates the targets to the outputs as shown in Figure 10. The correlation coefficient in this case was found to be 0.93788 which indicates satisfactory correlation between the targets and the outputs.

**Figure 11:**
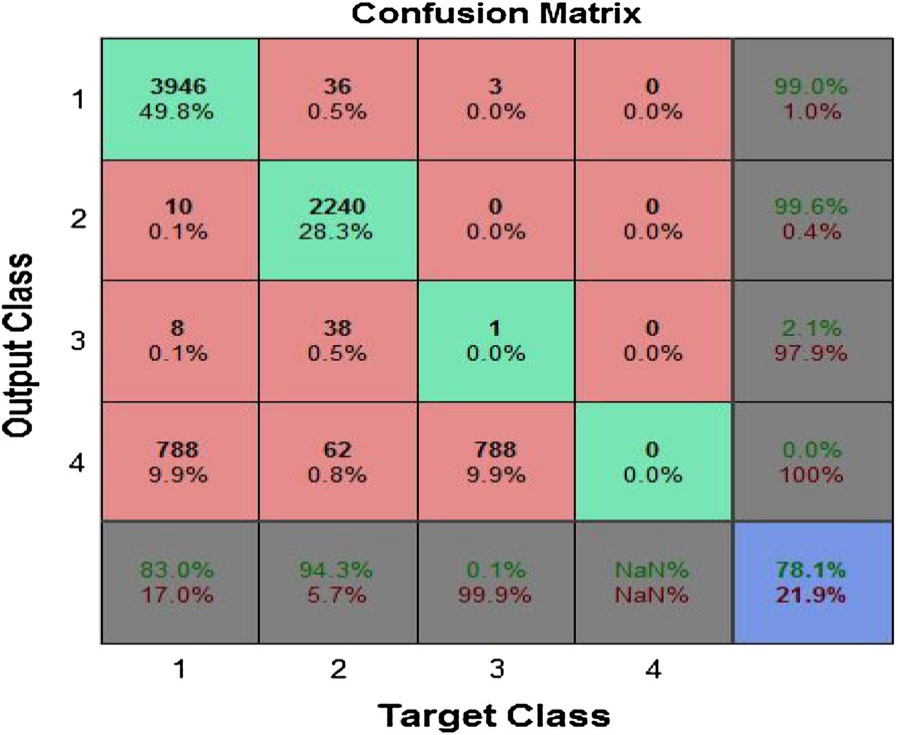
Confusion matrix for the training, validation and testing phases.

Figure 11 shows that the efficiency of the trained neural network in terms of its ability to check the type of the fault, which is 78.1 percent. It is concluded that the neural network can differentiate among the all ten possible types of faults on a transmission line.

## Conclusion

In this paper we have studied the application of artificial neural networks for the detection and classification of faults on a three phase transmission lines system. The method developed utilizes the three phase voltages and three phase currents as inputs to the neural networks. The inputs were normalized with respect to their pre-fault values respectively. The results shown in the paper is for line to ground fault only. The other types of faults, e.g. line-to-line, double line-to-ground and symmetrical three phase faults can be studied and ANNs can be developed for each of these faults.

All the artificial neural networks studied here adopted the back-propagation neural network architecture. The simulation results obtained prove that the satisfactory performance has been achieved by all of the proposed neural networks and are practically implementable. The importance of choosing the most appropriate ANN configuration, in order to get the best performance from the network, has been stressed upon in this work. The sampling frequency adopted for sampling the voltage and current waveforms in this research work is 1,000 Hz.

Some important conclusions that can be drawn from the research are:

1. Artificial neural networks are a reliable and effective method for an electrical power system transmission line fault classification and detection especially in view of the increasing dynamic connectivity of the modern electrical power transmission systems.
2. The performance of an artificial neural network should be analyzed properly and particular neural network structure and learning algorithm before choosing it for a practical application.
3. Back propagation neural networks delivers good performance, when they are trained with large training data set, which is easily available in power systems and hence back propagation networks have been chosen for proposed method.

The scope of ANN is wide enough and can be explored more. The fault detection and classification can be made intelligent by nature by developing suitable intelligent techniques. This can be achieved if we have the computers which can handle large amount of data and take least amount time for calculations.
